# Supplementary material for: From sequence to enzyme mechanism using multi-label machine learning
Source: BMC Bioinformatics. 2014 May 19;15:150. doi: 10.1186/1471-2105-15-150 (PMC4229970; doi:10.1186/1471-2105-15-150)
Supplement: Additional file 2 — Java code of ml2db. Additional file ml2db_code.tar.gz contains the Java source code to run the multi-label machine learning experiments and save the results to database. The code’s Javadoc is included. [file 1471-2105-15-150-S2.zip › additional file 2/ml2db/ml2db/doc/serialized-form.html]

Serialized Form


JavaScript is disabled on your browser.


- Overview
- Package
- Class
- Use
- Tree
- Deprecated
- Index
- Help

- Prev
- Next

- Frames
- No Frames

- All Classes

# Serialized Form

- ## Package main.ml2db

  - ### Class main.ml2db.DbManagers extends java.lang.Object implements Serializable

    serialVersionUID:
    :   2685002297805361491L

    - ### Serialized Fields

      - #### m\_dataDb

        ```
        uk.ac.standrews.utils.main.database.DbManager m_dataDb
        ```
      - #### m\_arffRecordsDb

        ```
        uk.ac.standrews.utils.main.database.DbManager m_arffRecordsDb
        ```
      - #### m\_mlResultsDb

        ```
        uk.ac.standrews.utils.main.database.DbManager m_mlResultsDb
        ```
      - #### m\_dataDbPropsFile

        ```
        java.lang.String m_dataDbPropsFile
        ```
      - #### m\_arffDbManagerPropsFile

        ```
        java.lang.String m_arffDbManagerPropsFile
        ```
      - #### m\_resultsDbManagerPropsFile

        ```
        java.lang.String m_resultsDbManagerPropsFile
        ```
- ## Package main.ml2db.al.utility.globaldistance

  - ### Class main.ml2db.al.utility.globaldistance.DistancesCalculator extends java.lang.Object implements Serializable

    serialVersionUID:
    :   -11955856628164450L

    - ### Serialized Fields

      - #### m\_filePath

        ```
        java.lang.String m_filePath
        ```
      - #### m\_debug

        ```
        boolean m_debug
        ```
      - #### m\_writeToFile

        ```
        boolean m_writeToFile
        ```

        whether to write instance1+instance2+distance to file
      - #### m\_instances

        ```
        IdMultiLabelInstances m_instances
        ```
      - #### m\_distanceFunction

        ```
        weka.core.NormalizableDistance m_distanceFunction
        ```
      - #### m\_storage

        ```
        DistancesStorage m_storage
        ```
      - #### m\_stringPool

        ```
        uk.ac.standrews.utils.main.stringutils.StringPool m_stringPool
        ```
      - #### m\_distancesFileName

        ```
        java.lang.String m_distancesFileName
        ```
  - ### Class main.ml2db.al.utility.globaldistance.DistancesStorage extends java.lang.Object implements Serializable

    serialVersionUID:
    :   6107209898643271505L

    - ### Serialized Fields

      - #### m\_distances

        ```
        java.util.HashMap<K,V> m_distances
        ```

        Stores the two instances as a string (instance 1 | instance 2)
      - #### m\_debug

        ```
        boolean m_debug
        ```
      - #### m\_test

        ```
        boolean m_test
        ```
- ## Package main.ml2db.al.utility.neighbours

  - ### Class main.ml2db.al.utility.neighbours.LinearMultiLabelNNSearch extends weka.core.neighboursearch.LinearNNSearch implements Serializable

    serialVersionUID:
    :   1L

    - ### Serialized Fields

      - #### m\_dontNormalize

        ```
        boolean m_dontNormalize
        ```
      - #### m\_numberOfLabels

        ```
        int m_numberOfLabels
        ```
      - #### m\_labelIndices

        ```
        int[] m_labelIndices
        ```
      - #### m\_debug

        ```
        boolean m_debug
        ```
  - ### Class main.ml2db.al.utility.neighbours.LinearNNSearchSkipInstanceItself extends LinearMultiLabelNNSearch implements Serializable

    serialVersionUID:
    :   1L

    - ### Serialized Fields

      - #### m\_debug

        ```
        boolean m_debug
        ```
  - ### Class main.ml2db.al.utility.neighbours.Neighbourhood extends IdInstance implements Serializable

    serialVersionUID:
    :   1L

    - ### Serialized Fields

      - #### m\_isNeededBy

        ```
        java.util.Vector<E> m_isNeededBy
        ```

        the instances that have this instance as nearest neighbour
      - #### m\_numberOfNearestNeighbours

        ```
        int m_numberOfNearestNeighbours
        ```

        how many nearest neighbours this instance has
      - #### m\_percentPotentiallyLabelled

        ```
        double m_percentPotentiallyLabelled
        ```

        how much of this instance is already known
      - #### m\_labelled

        ```
        boolean m_labelled
        ```

        wether this instance has been labelled and added to the labelled set
      - #### m\_neigboursNeeded

        ```
        java.util.Vector<E> m_neigboursNeeded
        ```

        the neighbours of this instance (the instances this instance needs)
      - #### m\_debug

        ```
        boolean m_debug
        ```
  - ### Class main.ml2db.al.utility.neighbours.NeighbourhoodNetwork extends UtilityMetric implements Serializable

    serialVersionUID:
    :   9120612664130288260L

    - ### Serialized Fields

      - #### m\_unlabelledInstances

        ```
        IdMultiLabelInstances m_unlabelledInstances
        ```
      - #### m\_instancesTree

        ```
        java.util.HashMap<K,V> m_instancesTree
        ```
      - #### m\_neighboursSearcher

        ```
        LinearNNSearchSkipInstanceItself m_neighboursSearcher
        ```
      - #### m\_debug

        ```
        boolean m_debug
        ```
- ## Package main.ml2db.mulan.classifier.lazy

  - ### Class main.ml2db.mulan.classifier.lazy.BRkNNFewInstances extends BRkNNVisible implements Serializable

    serialVersionUID:
    :   9093046195423186044L
  - ### Class main.ml2db.mulan.classifier.lazy.BRkNNVisible extends mulan.classifier.lazy.MultiLabelKNN implements Serializable

    serialVersionUID:
    :   -1315629879770614775L

    - ### Serialized Fields

      - #### m\_file

        ```
        java.io.File m_file
        ```
      - #### m\_debug

        ```
        boolean m_debug
        ```
      - #### random

        ```
        java.util.Random random
        ```
      - #### avgPredictedLabels

        ```
        int avgPredictedLabels
        ```

        Stores the average number of labels among the knn for each instance Used
        in BRkNN-b extension
      - #### cvMaxK

        ```
        int cvMaxK
        ```

        The value of kNN provided by the user. This may differ from
        numOfNeighbors if cross-validation is being used.
      - #### cvkSelection

        ```
        boolean cvkSelection
        ```

        Whether to select k by cross validation.
      - #### extension

        ```
        BRkNNVisible.ExtensionType extension
        ```

        The type of extension to be used
- ## Package main.ml2db.mulan.learn

  - ### Class main.ml2db.mulan.learn.MulanLearner extends java.lang.Object implements Serializable

    - ### Serialized Fields

      - #### m\_learner

        ```
        mulan.classifier.MultiLabelLearner m_learner
        ```
      - #### m\_learnerBaseType

        ```
        java.lang.String m_learnerBaseType
        ```
      - #### m\_learnerType

        ```
        java.lang.String m_learnerType
        ```
- ## Package main.ml2db.mulan.learn.evaluation

  - ### Class main.ml2db.mulan.learn.evaluation.FalseNegatives extends MicroFMeasurePrinter implements Serializable

    serialVersionUID:
    :   -1953293390147659720L
  - ### Class main.ml2db.mulan.learn.evaluation.FalsePositives extends MicroFMeasurePrinter implements Serializable

    serialVersionUID:
    :   -1953293390147659720L
  - ### Class main.ml2db.mulan.learn.evaluation.MicroFMeasurePrinter extends mulan.evaluation.measure.MicroFMeasure implements Serializable

    serialVersionUID:
    :   1L

    - ### Serialized Fields

      - #### m\_debug

        ```
        boolean m_debug
        ```
  - ### Class main.ml2db.mulan.learn.evaluation.TruePositives extends MicroFMeasurePrinter implements Serializable

    serialVersionUID:
    :   -1953293390147659720L
- ## Package main.ml2db.mulan.mulanarff

  - ### Class main.ml2db.mulan.mulanarff.IdInstance extends weka.core.SparseInstance implements Serializable

    serialVersionUID:
    :   247791787100877093L

    - ### Serialized Fields

      - #### m\_debug

        ```
        boolean m_debug
        ```
      - #### m\_identifier

        ```
        java.lang.String m_identifier
        ```
      - #### m\_wekaInstance

        ```
        weka.core.Instance m_wekaInstance
        ```
      - #### m\_string

        ```
        java.lang.String m_string
        ```
  - ### Class main.ml2db.mulan.mulanarff.IdMultiLabelInstances extends mulan.data.MultiLabelInstances implements Serializable

    serialVersionUID:
    :   8065183979298605452L

    - ### Serialized Fields

      - #### m\_id2Instances

        ```
        java.util.TreeMap<K,V> m_id2Instances
        ```
      - #### m\_id2IdInstances

        ```
        java.util.TreeMap<K,V> m_id2IdInstances
        ```
      - #### m\_instanceNames2AttributesIndexStringMap

        ```
        java.util.TreeMap<K,V> m_instanceNames2AttributesIndexStringMap
        ```
      - #### m\_instanceNames2AttributesNamesStringMap

        ```
        java.util.TreeMap<K,V> m_instanceNames2AttributesNamesStringMap
        ```
      - #### m\_instNames2SparseAttrStringMap

        ```
        java.util.TreeMap<K,V> m_instNames2SparseAttrStringMap
        ```
      - #### m\_arffId

        ```
        int m_arffId
        ```
      - #### m\_debug

        ```
        boolean m_debug
        ```
      - #### m\_dbMan

        ```
        DbManagers m_dbMan
        ```
      - #### m\_arffFilePath

        ```
        java.lang.String m_arffFilePath
        ```
      - #### m\_xmlFilePath

        ```
        java.lang.String m_xmlFilePath
        ```
      - #### m\_attributesStringPool

        ```
        uk.ac.standrews.utils.main.stringutils.StringPool m_attributesStringPool
        ```
      - #### m\_instanceNamesStringPool

        ```
        uk.ac.standrews.utils.main.stringutils.StringPool m_instanceNamesStringPool
        ```
      - #### m\_labelNames

        ```
        java.lang.String[] m_labelNames
        ```
      - #### m\_labelsToPartition

        ```
        java.util.TreeMap<K,V> m_labelsToPartition
        ```
      - #### m\_instance2LabelsPartition

        ```
        java.util.TreeMap<K,V> m_instance2LabelsPartition
        ```

        for each instance, the partition of label attribute (true if class label
        is present, false if absent)
      - #### m\_toBeProcessed

        ```
        java.util.Set<E> m_toBeProcessed
        ```

        the instances not yet predicted
      - #### m\_featureAttributes

        ```
        java.util.Set<E> m_featureAttributes
        ```
      - #### m\_classAttributes

        ```
        java.util.Set<E> m_classAttributes
        ```
      - #### m\_logFile

        ```
        java.io.File m_logFile
        ```
      - #### m\_wekaInstString2IdInst

        ```
        uk.ac.standrews.utils.main.maputils.OneToManyMap<T,U> m_wekaInstString2IdInst
        ```
      - #### m\_instanceStringPool

        ```
        uk.ac.standrews.utils.main.stringutils.StringPool m_instanceStringPool
        ```
      - #### m\_instString2LabelsString

        ```
        java.util.TreeMap<K,V> m_instString2LabelsString
        ```
  - ### Class main.ml2db.mulan.mulanarff.Statistics extends java.lang.Object implements Serializable

    serialVersionUID:
    :   1206845794397561633L

    - ### Serialized Fields

      - #### m\_debug

        ```
        boolean m_debug
        ```
      - #### numInstances

        ```
        int numInstances
        ```

        the number of instances
      - #### numPredictors

        ```
        int numPredictors
        ```

        the number of predictive attributes
      - #### numNominal

        ```
        int numNominal
        ```

        the number of nominal predictive attributes
      - #### numNumeric

        ```
        int numNumeric
        ```

        the number of numeric attributes
      - #### numLabels

        ```
        int numLabels
        ```

        the number of labels
      - #### labelDensity

        ```
        double labelDensity
        ```

        the label density
      - #### labelCardinality

        ```
        double labelCardinality
        ```

        the label cardinality
      - #### examplesPerLabel

        ```
        double[] examplesPerLabel
        ```

        percentage of instances per label
      - #### numClassLabelsWithExamples

        ```
        int numClassLabelsWithExamples
        ```

        the number of class labels having at least one example
      - #### numAttributesWithExamples

        ```
        int numAttributesWithExamples
        ```

        the number of attributes having at least one example
      - #### cardinalityDistribution

        ```
        double[] cardinalityDistribution
        ```

        number of examples per cardinality,   
          
        note that this array has size equal to the number of elements plus one,   
        because the first element is the number of examples for cardinality=0
      - #### labelsets

        ```
        java.util.HashMap<K,V> labelsets
        ```

        labelsets and their frequency
      - #### phi

        ```
        double[][] phi
        ```

        the array holding the phi correlations
- ## Package main.ml2db.weka

  - ### Class main.ml2db.weka.IdentifiedSparseInstance extends weka.core.SparseInstance implements Serializable
  - ### Class main.ml2db.weka.InstAttValueMap extends uk.ac.standrews.utils.main.maputils.MapOfMaps implements Serializable

    serialVersionUID:
    :   6823379379901766471L

    - ### Serialized Fields

      - #### m\_attributeType

        ```
        java.lang.String m_attributeType
        ```
      - #### m\_feature\_or\_class\_attribute

        ```
        java.lang.String m_feature_or_class_attribute
        ```
  - ### Class main.ml2db.weka.JaccardDistance extends weka.core.NormalizableDistance implements Serializable

    serialVersionUID:
    :   241889613095897453L

    - ### Serialized Fields

      - #### m\_debug

        ```
        boolean m_debug
        ```
  - ### Class main.ml2db.weka.JaccardDistanceStored extends java.lang.Object implements Serializable

    serialVersionUID:
    :   -2402319683009190551L

    - ### Serialized Fields

      - #### m\_jaccardDistanceStore

        ```
        DistancesStorage m_jaccardDistanceStore
        ```

        Stores the Jaccard distance for instance A and instance B. Jaccard
        distance = 1 minus (intersection size divided by union size).
      - #### m\_idInstances

        ```
        IdMultiLabelInstances m_idInstances
        ```
      - #### m\_instance2NonZeroFeatAttIndexes

        ```
        java.util.TreeMap<K,V> m_instance2NonZeroFeatAttIndexes
        ```
      - #### m\_instances

        ```
        weka.core.Instances m_instances
        ```
      - #### m\_featAttributes

        ```
        java.util.HashSet<E> m_featAttributes
        ```
      - #### m\_debug

        ```
        boolean m_debug
        ```
  - ### Class main.ml2db.weka.JaccardEuclideanDistance extends JaccardDistance implements Serializable

    serialVersionUID:
    :   1057726876137658605L

    - ### Serialized Fields

      - #### m\_euclideanDistance

        ```
        weka.core.EuclideanDistance m_euclideanDistance
        ```
- ## Package test.ml2db.mulan.learn.traintest

  - ### Class test.ml2db.mulan.learn.traintest.TrainTestEvaluatorSerialisedTest extends java.lang.Object implements Serializable
  - ### Class test.ml2db.mulan.learn.traintest.TrainTestExpSerializedTest extends java.lang.Object implements Serializable

- Overview
- Package
- Class
- Use
- Tree
- Deprecated
- Index
- Help

- Prev
- Next

- Frames
- No Frames

- All Classes
